# Neobaicalein prevents isoflurane anesthesia-induced cognitive impairment in neonatal mice via regulating CREB1

**DOI:** 10.1016/j.clinsp.2023.100201

**Published:** 2023-04-28

**Authors:** Niming Wu, Hua Liu, Xiang Lv, Yu Sun, Hong Jiang

**Affiliations:** Department of Anesthesiology, Shanghai Ninth People's Hospital, Shanghai Jiao Tong University School of Medicine, Shanghai, China

**Keywords:** Neobaicalein, Isoflurane, Neuroinflammatory, CREB1, Cognitive impairment

## Abstract

**Objectives:**

Isoflurane (ISO) is widely used in the clinic and research. The authors aimed to explore whether Neobaicalein (Neob) could protect neonatal mice from ISO-induced cognitive damage.

**Method:**

The open field test, Morris water maze test, and tail suspension test was performed to assess the cognitive function in mice. Enzyme-linked immunosorbent assay was used to evaluate inflammatory-related protein concentrations. Immunohistochemistry was used to assess Ionized calcium-Binding Adapter molecule-1 (IBA-1) expression. Hippocampal neuron viability was detected using the Cell Counting Kit-8 assay. Double immunofluorescence staining was employed to confirm the interaction between proteins. Western blotting was used to assess protein expression levels.

**Results:**

Neob notably improved cognitive function and exhibited anti-inflammatory effects; moreover, under iso-treatment, it exhibited neuroprotective effects. Furthermore, Neob suppressed interleukin-1β, tumor necrosis factor-α, and interleukin-6 levels and upregulated interleukin-10 levels in ISO-treated mice. Neob significantly mitigated iso-induced increases in IBA-1–positive cell numbers of the hippocampus in neonatal mice. Furthermore, it inhibited ISO-induced neuronal apoptosis. Mechanistically, Neob was observed to upregulate cAMP Response Element Binding protein (CREB1) phosphorylation and protected hippocampal neurons from ISO-mediated apoptosis. Moreover, it rescued ISO-induced abnormalities of synaptic protein.

**Conclusions:**

Neob prevented ISO anesthesia-induced cognitive impairment by suppressing apoptosis and inflammation through upregulating CREB1.

## Highlights

• Neobaicalein ameliorates isoflurane-induced cognitive impairment in neonatal mice.
• Neobaicalein reduces isoflurane treatment-induced inflammation.
• Neobaicalein reduces isoflurane-induced apoptosis of hippocampal neurons through the p-CREB1 pathway.

## Introduction

The reverse effects of general anesthetics on the neuronal system have been widely recognized recently. Accumulating studies have proven that early general anesthetics exposure damaged neuronal system development by suppressing neurogenesis and damaging long-term neurocognitive function.^1^, ^2^, ^3^ It has been reported that exposure to general anesthesia may lead to potential neurocognitive damage in young children.^4^^,^^5^ Recently, several studies have focused on the adverse effects of anesthesia. Potential protective factors for neuronal damage, which was induced by general anesthesia, are worth exploring and developing.

Isoflurane (ISO) is widely used in general anesthesia in the clinic because of its excellent sedative and analgesic effects.^6^ It was reported that ISO could lead to more apoptosis than sevoflurane.^7^ Furthermore, further ISO exposure was associated with long-term memory loss.^8^ Furthermore, ISO was reported to inhibit neurogenesis, promote neuroapoptosis, and stimulate neural damage, thereby leading to long-term neurocognitive and memory impairment during brain development.^9^, ^10^, ^11^ Therefore, it is necessary to develop protective effectors to inhibit the reverse effects of ISO in the clinic.

Neobaicalein (Skullcapflavone II) (Neob), an active compound, is extracted from the roots of *Scutellaria pinnatifida*.^12^, ^13^, ^14^ Parsafar, S., et al. confirmed that Neob protected the neurons from rotenone-induced neurotoxicity and inhibited inflammation under Lipopolysaccharide (LPS) treatment.^15^^,^^16^ Additionally, Neob could increase antioxidant activity.^14^ Moreover, accumulating studies indicated that Neob had remediation activity in several diseases. It inhibited the function of human skin fibroblasts by promoting type I collagen degradation, which damaged the integrity of the extracellular matrix and prevented airway inflammation in an asthma mouse model.^16^^,^^17^ The correlation between Neob and its neuroprotective effect remains unclear. cAMP Response Element Binding protein (CREB)^18^ is a member of the basic-region leucine zipper superfamily. CREB could activate several gene expressions, which are important for neuronal survival. CREB1 is primarily known for its role in neurons.^19^, ^20^, ^21^ It has been shown that CREB-related genes are dysregulated in the brain of a patient with Alzheimer's disease as manifested by reduced levels of CREB-regulated brain-derived neurotrophic factor.^22^, ^23^, ^24^ It has been reported that this effect may be associated with cognitive decline. Studies have reported the relationship between Neob and CREB;^18^ however, whether Neob can affect the cognitive ability of neonatal mice through the same pathway as CREB also needed to be explored.

Here, the authors aimed to verify whether Neob improved neurocognitive deficits following exposure to ISO. The authors further explored whether CREB1 mediated the neuroprotective effect of Neob in mice.

## Materials and methods

### Animals’ experimental design

Animal experiments were approved by the Ethical Committee on Animal Experimentation of Shanghai Ninth People's Hospital, Shanghai, China (nº SH9H-2022-A543-SB). All experimental procedures were performed following the Guide for the National Science Council of the Republic of China. All experimental procedures complied with the ARRIVE guidelines.

Mice used in the present study were postnatal day 7 C57BL/6 since they were most susceptible to neuronal insult induced by general anesthetics.^25^ Mice were purchased from Shanghai Laboratory Animal Research Center and cross-fostered and housed with the following standard conditions: room temperature, 23±1°C; relative humidity, 60%±5%; and a 2h light/dark cycle.

All mice were randomly divided into the following three groups: control, ISO, and ISO+Neob. Mice in the ISO+Neob group received a dose of 100 mg/kg Neob by intragastric administration for 7 days after exposure to ISO anesthesia. Mice in the control and ISO groups received an identical volume of saline containing 0.25% DMSO by intragastric administration. The ISO anesthesia procedure was performed following previous protocols.^26^ Briefly, mice in the ISO and Iso+Neob groups were exposed to 2% ISO for 2h, whereas mice in the control group received a vehicle gas (40% O_2_ + 60% N_2_). Mice were put in anesthesia-induction chambers that were kept in a homeothermic incubator (33°C). Following anesthesia, all mice were sent back to dams until they were fully awake. Twelve hours following anesthesia ended, fifteen rats in each group were randomized to be sacrificed, and the hippocampi were removed for Enzyme-Linked Immunosorbent Assay (ELISA) (n=5), Immunohistochemistry (IHC) (n=5), immunofluorescence (n=5), and western blotting (n=5). The remaining mice (n=10) were used for the Open Field Test (OFT), Morris Water Maze (MWM) test, and Tail Suspension Test (TST) test.

### OFT

The bottom of the chamber was virtually divided into nine squares (10×10 cm). Mice were placed onto the central square and allowed to explore the maze freely for 5 min. OFT was employed to evaluate the cognitive impairment of mice by assessing the time of staying in the central area, the time of entering the central area, and the total moving distance.

### MWM test

MWM test was performed as previously reported.^27^ Briefly, a platform (approximately 10 cm diameter) was submerged in a circular tank (120 cm diameter; 50 cm high) filled with warm (22°C) opaque water mixed homogeneously with carbonic ink. Training would last for 4 days twice daily. Each part was comprised of three times of trials. Before each trial, mice were released from assigned quadrants that did not contain the platform in the tank. Each mouse was provided 60s to locate the hidden platform and stay on the platform for 15s after it was located. If mice could not locate the hidden platform in the ruled time, they would be guided to the platform and removed from the platform 15s later. The time for locating the platform (latency) was recorded and analyzed.

A probe trial without the platform was performed to evaluate memory retention for the hidden platform location on the fifth day. Mice were placed in a quadrant that did not contain the platform and allowed to swim freely for 60s. The percentage of time staying in the target quadrant was recorded and considered an indicator of memory retention.

### TST

Mice were suspended 20 cm above the floor using medical tape placed approximately 1 cm from the tip of the tail in a quiet environment. Subsequently, they were suspended for 2 min for acclimation followed by 4 min for detection. The immobility time (s) of mice in the last 4 min was recorded.

### Treatment of animal hippocampus sample

The mice were sacrificed following all experiments. The whole brain of the mice was removed on ice and placed into pre-cooled Phosphate-Buffered Saline (PBS); subsequently, the hippocampi were removed and stored at -80°C for further study.

### ELISA

ELISA was employed to detect Interleukin (IL)-1β, Tumor Necrosis Factor (TNF)-α, and interleukin-6, IL-6, and IL-10 concentrations using a rat IL-1β, TNF-α, IL-6, and IL-10 ELISA kit (R&D Systems, Inc., Minneapolis, MN, USA) following the manufacturer's instruction.

### Western blotting

The total protein was extracted using a radioimmunoprecipitation assay, and the bicinchoninic acid (Beyotime, Shanghai, China) method was employed to quantify the concentration. The protein sample was first electrophoresed for 2h; subsequently, the authors transferred the total protein onto polyvinylidene difluoride membranes (Millipore, Billerica, MA, USA). TBST containing 5% skim milk was used to block non-specific antigens for 1h following incubating with primary antibodies (caspase-3 [1:1,000, ab32351; Abcam], c-caspase-3 [1:1,000, ab32042; Abcam], CREB1 [1:1,000, ab32515; Abcam], p-CREB1 [1:1,000, ab32096; Abcam], Bcl-2 [1:1,000, ab182858; Abcam], PSD-95 [1:1,000, ab238135; Abcam], synaptophysin [1:1,000, ab32127; Abcam], synapsin [1:1,000, ab254349; Abcam], GAPDH [1:1,000, ab8245; Abcam], and β-actin [1:5,000, #A5441, Sigma]) at 4°C overnight. Membranes were washed with TBST three times and incubated with the secondary HRP-conjugated goat anti-rabbit IgG (1:5,000 dilution; cat. ab205719; Abcam). Subsequently, membranes were washed with TBST three times. Blots were then visualized using enhanced chemiluminescence (GE Healthcare Life Sciences, Little Chalfont, UK).

### Primary neuronal culture

Mice hippocampal neurons were isolated from P0 mice and subsequently cryopreserved at the primary passage. All samples were stained positive for PGP and Tuj-1 and tested negative for mycoplasma. All procedures, including medium preparation, cell culture plate coating, thawing of cells/initiation of culture process, and cell culture maintenance, were performed following the manufacturer's instructions. Cells were seeded for experiments and underwent ISO or ISO+Neob (150 μM) (ISO+Neob) exposures for 0h, 3h, or 6h. Primary neuronal cell morphology was assessed daily by microscopy.

### Cell Counting Kit-8 (CCK-8) assay

Approximately 1,500 cells were seeded into 96-well plates and cultured for the indicated days. CCK-8 solution and medium (1:10) and incubated at 37°C for 2h. The absorbance was detected at 450 nm by using a microplate reader (Bio-Rad 680, Bio-Rad, Hercules, CA, USA).

### Immunofluorescence

Briefly, PBS was used to wash the slides three times, which were fixed in 4% paraformaldehyde. Ten minutes later, 0.1% Triton X-100 in PBS was employed to permeabilize cells for 15 min. Then, tissues were blocked with 2% BSA in TBST for 1h. The antibody (NeuN [1:400, cat. No. ABN78; Millipore], p-CREB1 [1:200, cat. No. ab32096; Abcam], β3-Tubulin [1:400, cat. No. MAB1637; Millipore], and caspase-3 [1:100, cat. No. sc-7272; Santa Cruz]) was incubated overnight, and the second antibody was incubated for 1h. DAPI was used to stain the nuclei. Subsequently, the images were photographed using OLYMPUS FV1000.

### Immunohistochemical staining

Briefly, tissues were deparaffinized with xylene and rehydrated in a graded ethanol series; the slides were incubated with 3% (v/v) hydrogen peroxide followed by antigen retrieval in the citrate-mediated high-temperature. Subsequently, slides were incubated with the primary antibodies (Ionized calcium-Binding Adapter molecule-1 [IBA-1]) (1:200, cat. No. ab178846; Abcam) at 4°C overnight, followed by secondary antibody incubation for 1h at 25°C, and stained with diaminobenzidine. Images were photographed using a microscope.

### Statistical analysis

Data were expressed as means ± SEM. The Bartlett test was used to determine whether the present data were standard normal distributions. A two-sided analysis of variance or *t*-test was used to assess differences for standard normal distribution data. A p-value of <0.05 was considered statistically significant.

## Results

### Neob ameliorates ISO-induced cognitive impairment in neonatal mice

**Fig. 1:**
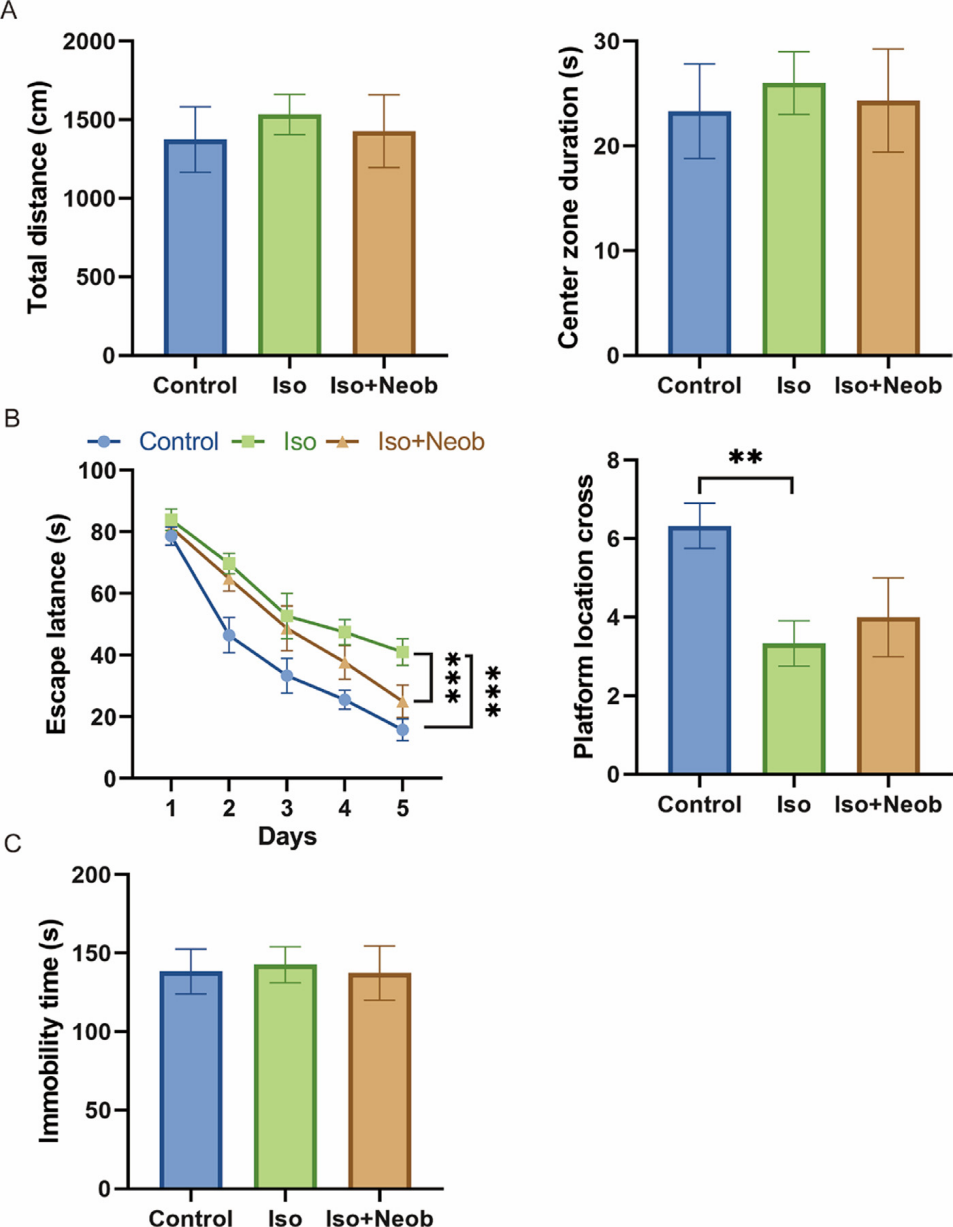
Neob improves ISO-induced cognitive impairment. (A) The total distance and time of mice staying in the central area in each group are recorded in the open field test. (B) Effects of Neob on memory and spatial learning functions in mice evaluated using the Morris water maze test. (C) Immobility time of the mice is evaluated using the tail suspension test.

First, the authors examined the effects of ISO on neonatal mice; data from the OFT showed that ISO did not affect depressive/anxiety-like behavior in mice (Fig. 1A). Data from the MWM indicated that ISO treatment remarkably increased the escape latency of mice as well as impaired mice crossing of the platform, indicating that ISO indeed led to cognitive impairment in neonatal mice, whereas Neob treatment significantly alleviated ISO-induced cognitive impairment (Fig. 1B). Tail suspension experiments showed that ISO treatment did not induce a depressive state in mice (Fig. 1C). Collectively, these results show that Neob attenuates ISO treatment-induced cognitive impairment in neonatal mice.

### Neob reduces the elevated level of ISO treatment-induced inflammation

**Fig. 2:**
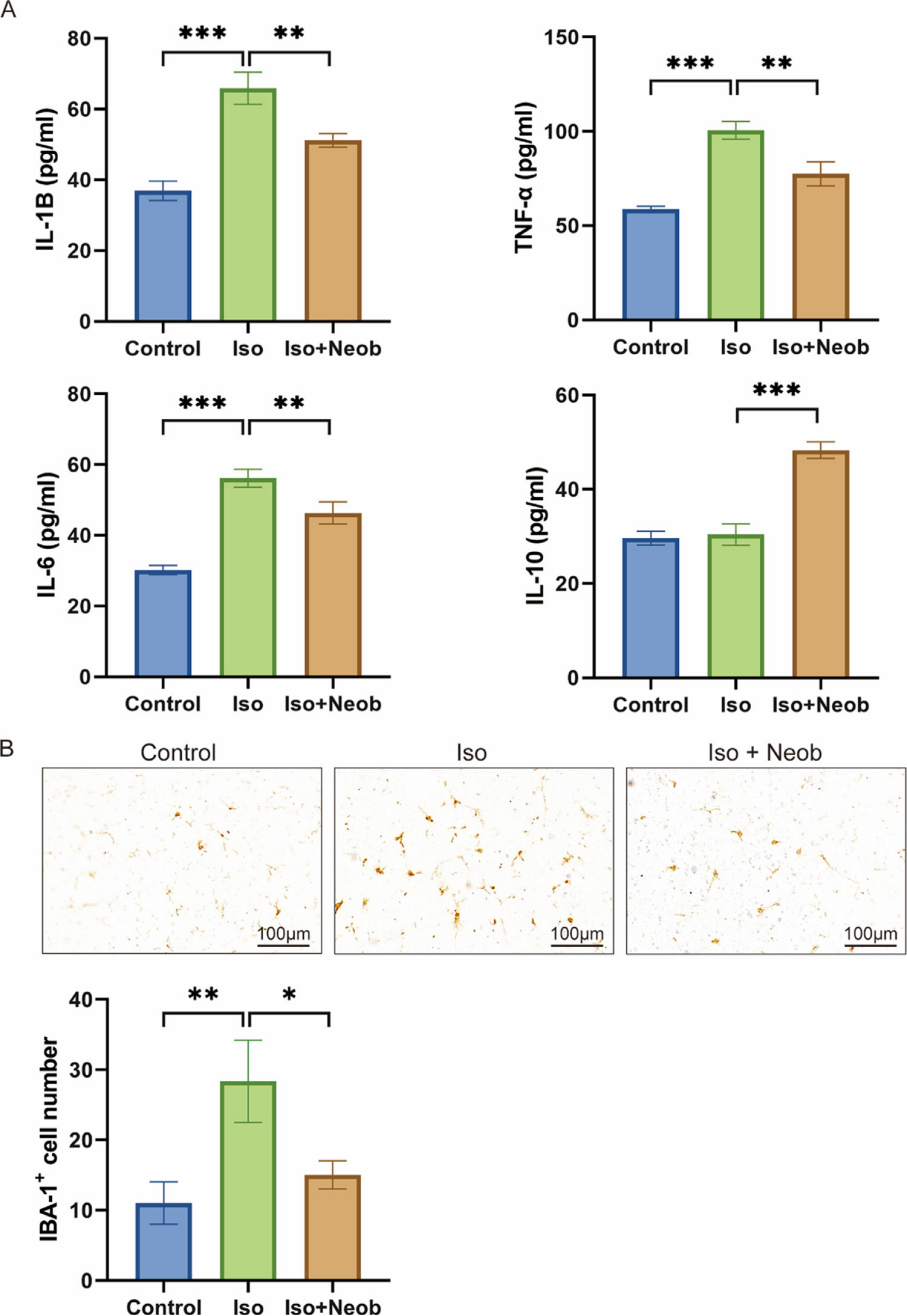
Neob reduces ISO treatment-induced elevated levels of inflammation. (A) ELISA assay is used to detect the IL-1b, TNF-a, IL-6, and IL-10 concentrations in the mice serum in each group. (B) IHC detection of the ratio of IBA-1–positive cells in mice brain sections in each group.

Next, the authors explored the concentrations of inflammatory-associated proteins in the hippocampus of neonatal mice induced by ISO treatment. ELISA data showed that ISO exposure significantly increased IL-1β, TNF-α, and IL-6 concentrations in the hippocampus compared with the control condition. However, Neob treatment notably inhibited the production of several inflammatory cytokines in the hippocampus of mice (Fig. 2A). Simultaneously, Neob treatment upregulated the IL-10 expression, suggesting its anti-inflammatory effect (Fig. 2A). To further investigate the inhibitory effect of Neob treatment on ISO-mediated inflammation levels, the authors examined the activation of microglia following Neob treatment for 7 days. IHC indicated that ISO markedly activated the expression of the microglial marker, IBA-1, whereas Neob treatment inhibited this effect, suggesting that Neob treatment suppressed the inflammatory response in the brain **(**Fig. 2B). These results suggest that Neob mitigates the elevated level of ISO treatment-induced inflammation effect in the hippocampus of neonatal mice.

### Neob treatment reduces ISO-mediated neuronal apoptosis

**Fig. 3:**
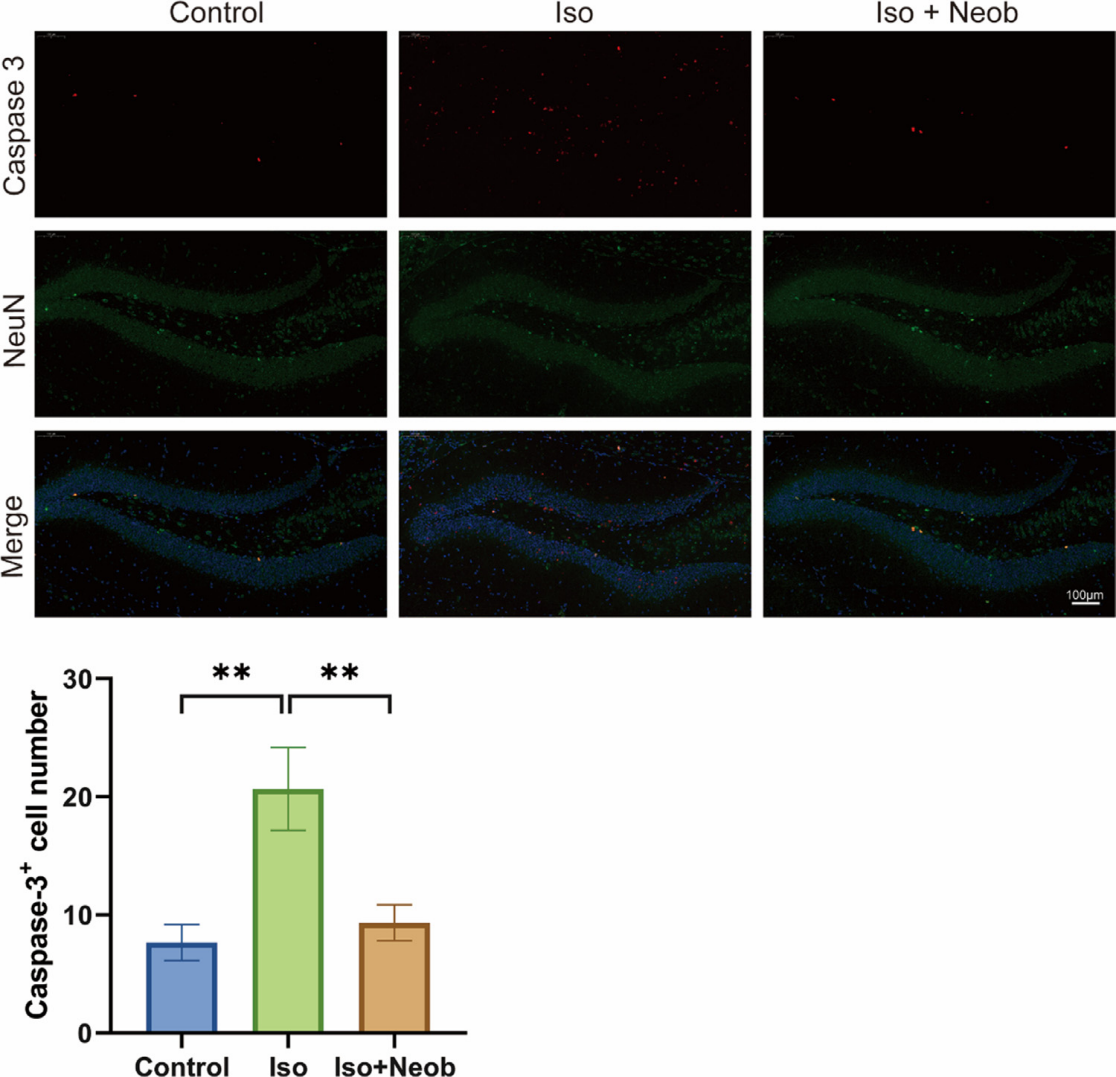
Neob treatment reduces ISO-mediated neuronal apoptosis. After treatment with ISO and Neob, the mice are sacrificed for immunofluorescence, labeled with caspase-3 (red), and NeuN (green), and the colocalization in the hippocampus is observed and statistically analyzed.

The authors further investigated whether ISO-mediated cognitive impairment is related to neuronal apoptosis. As shown in Fig. 3A, immunofluorescence staining of mouse brains marks the expression of caspase-3 (red) and NeuN (green). Colocalization analysis revealed that ISO exposure caused a remarkable increase in the proportion of apoptosis in NeuN-positive cells, whereas Neob treatment decreased this proportion, suggesting that ISO-induced neuronal apoptosis, whereas Neob treatment inhibited neuronal apoptosis.

### Neob reduces iso-induced apoptosis of hippocampal neurons through the p-CREB1 pathway

**Fig. 4:**
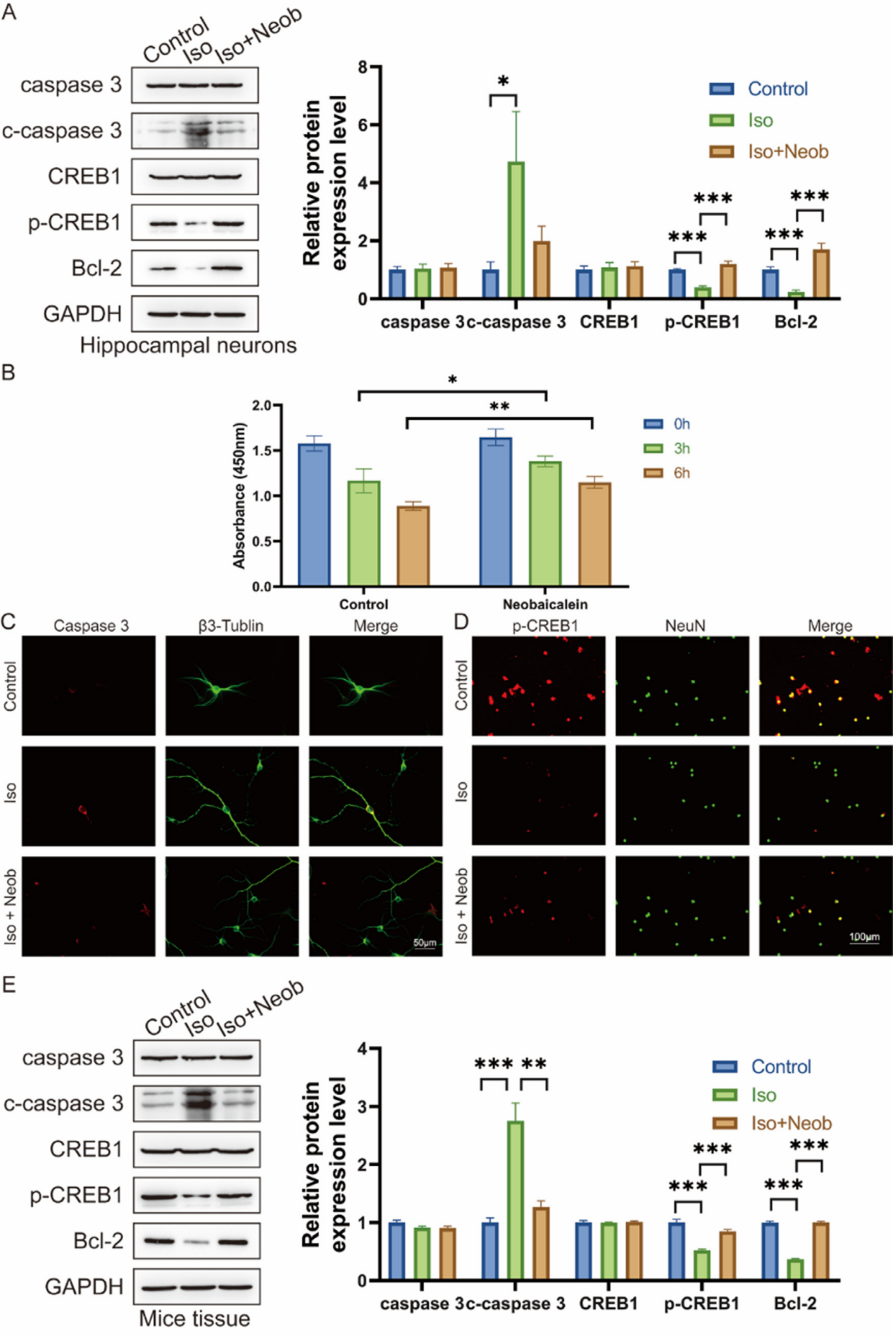
Neob reduces iso-induced apoptosis of hippocampal neurons via the p-CREB1 pathway. (A) Neural stem cells are treated with Neob and subsequently injected with ISO. Apoptosis and CREB1 phosphorylation levels are evaluated using western blot. (B) Primary hippocampal neurons are treated with Neob and subsequently injected with ISO for 0h, 3h, and 6h. Next, the cell viability of hippocampal neurons is evaluated using the CCK-8 assay. (C) Immunofluorescence of primary hippocampal neurons using caspase-3 (red) and β3-Tubulin (green) to label cells. (D) IF of primary hippocampal neurons using p-CREB1 (red) and NeuN (green) to label cells. (E) Caspase-3, cleaved caspase-3, CREB1, p-CREB1, and Bcl-2 expression in mouse brain tissue are evaluated using the western blot assay.

To explore the mechanism of the protective effects of Neob, the authors cultured hippocampal neurons in vitro. Following in vitro intervention with ISO and Neob, ISO treatment notably increased caspase-3, cleaved caspase-3 level, and decreased Bcl-2 expression, whereas Neob treatment alleviated the effects of ISO and upregulated CREB1 phosphorylation (Fig. 4A). CCK-8 data indicated that Neob treatment protected neurons from Neob-mediated reduction in viability (Fig. 4B). In an in vitro co-labeling experiment of caspase-3 and β3-Tubulin, the authors noted that Neob treatment protected cells from ISO-mediated apoptosis (Fig. 4C) and upregulated CREB1 phosphate in the NeuN-positive cell level (Fig. 4D). Neob reversed the biological effect of ISO, whereas studies of the neonatal mouse brain tissue showed that ISO inhalation resulted in caspase-3 upregulation, cleaved caspase-3 in the brain, and decreased CREB1 phosphorylation levels and the Bcl2 expression effect (Fig. 4E).

### Neob rescues ISO-induced abnormalities of synaptic proteins

**Fig. 5:**
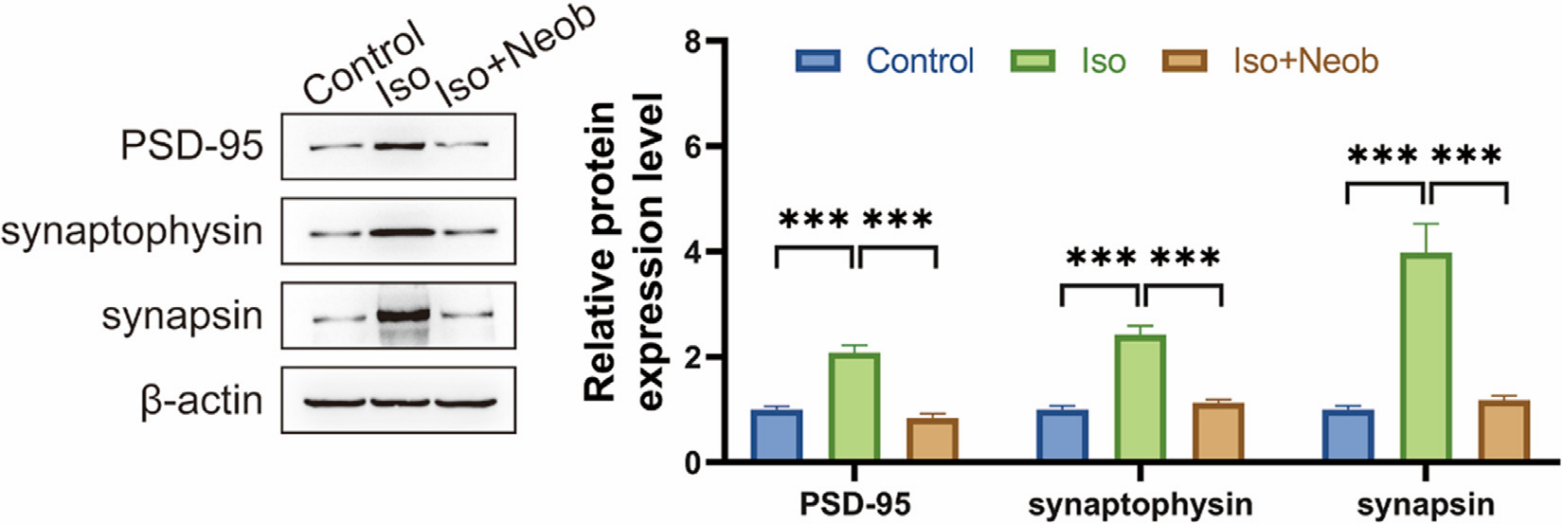
Neob rescues ISO-induced abnormalities of synaptic protein. After neonatal mice are treated with Neob and inhaled with ISO, the PSD-95, synaptophysin, and synasin expressions in mice brains are detected using western blot.

Cognitive impairment is frequently related to abnormalities in synaptic connections. The authors subsequently investigated whether inhaled ISO-induced cognitive impairment was associated with synaptic protein abnormalities. Western blot data showed that ISO inhalation significantly upregulated the expression of PSD-95, synaptophysin, and synapsin, suggesting that ISO caused abnormal synaptic protein expression, whereas Neob treatment reversed the effect of ISO on abnormal synaptophysin expression, suggesting the protective effect of Neob on synapse formation (Fig. 5).

## Discussion

With the increased exposure of neonates to anesthetics during central nervous system development, the association between adverse effects and brain damage had attracted more attention.^28^^,^^29^ Accumulating evidence proved that ISO may impair the neuronal system during brain development by inducing neuronal apoptosis,^30^, ^31^, ^32^, ^33^ which led to long-term cognitive impairment.^34^^,^^35^ Furthermore, ISO was proven to suppress the neurogenesis of rat brains.^36^^,^^37^ Zuo et al.^35^ reported that ISO treatment caused learning and memory function impairment by inhibiting the growth and differentiation of neural stem cells, thereby upregulating inflammation. Thus, the underlying mechanisms and protective factors for the adverse effects of ISO are worth exploring. Moreover, ISO-induced neurogenetic damage is poorly understood and explored.

Here, the authors observed that ISO treatment in neonatal mice resulted in learning and memory function impairment. Additionally, it has been proved that neonatal mice exposure to ISO led to neurogenetic damage, as proved by elevated PSD-95^38^ and synaptophysin levels in a previous study.^39^ This may underlie the cognitive impairment observed in the neonatal mouse brain. The present data demonstrated that ISO could induce fetal inflammation as evidenced by the increased expression of inflammatory-related proteins and caspase-3. Overall, the authors observed that ISO treatment in neonatal mice induces neuroinflammation and neurotoxicity, thereby leading to abnormal synuclein expression, which caused cognitive impairment in offspring mice.

Neob is an active compound derived from the root of *Scutellaria baicalensis*. Neob was not only protective against rotenone-induced neurotoxicity^14^ but also inhibited inflammatory responses when stimulated with LPS in glial cell cultures.^14^ Neob has high antioxidant activity and no obvious toxicity.^40^ However, the underlying mechanism of its neuroprotective effect remains understudied. Neob pretreatment showed neuroprotective effects, as manifested in the attenuating ISO-induced expression of inflammatory-related proteins and synaptophysin in neonatal mice and attenuating changes in cognitive impairment in neonatal mice. Thus, Neob exhibited neuroprotective effects by suppressing ISO-induced inflammation, rescuing at the synaptic level, and improving cognitive impairment. The present findings may have clinical implications for anesthetic exposure in neonates.

One explanation for the ISO-induced decreased CREB activity is the direct manipulation of its activity by histone deacetylase-4;^41^ In fact, the phosphorylation level of CREB1 was notably inhibited following ISO administration. The effects of the CREB family on neuronal system damage have been well explored. Moreover, the CREB family could support neuronal survival, regulate neuronal migration, modulate synaptogenesis, and promote long-term potentiation and memory formation. CREB1 is primarily known for its role in neurons; it is reported that CREB1 is active and plays a significant role in epilepsy. In patients with epilepsy, CREB1 is activated, and the related gene expression is upregulated.^42^ It has been reported that CREB1 activation and downstream gene expression promote the development of epilepsy in several models.^43^ Furthermore, lower CREB1 levels were correlated with a lower occurrence of spontaneous seizures.

Herein, the authors showed that Neob can directly increase the phosphorylation level of CREB1 and thus reverse the effects of ISO treatment. However, further studies in animals and humans are needed to confirm whether Neob alleviates anesthetic exposure-induced brain damage.

## Conclusion

Neob treatment attenuated ISO-induced brain inflammation as well as synaptophysin in neonatal mice by upregulating the CREB1 phosphorylation level, thereby alleviating cognitive impairment in neonatal mice. These findings may have clinical implications for anesthetic exposure in neonates.

## Authors’ contributions

Niming Wu and Hong Jiang designed experiments. Hua Liu, Xiang Lv and Yu Sun carried out experiments, and analyzed experimental results. Niming Wu wrote the manuscript. Hong Jiang revised the manuscript. All authors approved the final manuscript.

## Funding

This work was supported by the National Science Foundation under grant nº 82071177.

## Conflicts of interest

The authors declare no have conflicts of interest.
